# Immunohistological Analysis of * ABCD3* Expression in Caucasian and African American Prostate Tumors

**DOI:** 10.1155/2015/132981

**Published:** 2015-01-31

**Authors:** R. Renee Reams, Jacqueline Jones-Triche, Owen T. M. Chan, Brenda Y. Hernandez, Karam F. A. Soliman, Clayton Yates

**Affiliations:** ^1^College of Pharmacy & Pharmaceutical Sciences, Florida A&M University, Tallahassee, FL 32307, USA; ^2^Department of Biology and Environmental Sciences, Troy University, Troy, AL 36082, USA; ^3^Cancer Epidemiology, University of Hawaii Cancer Center, Honolulu, HI 96813, USA; ^4^Department of Biology and Center for Cancer Research, Tuskegee University, Tuskegee, AL 36088, USA

## Abstract

In a previously published study, we showed that expression of the* ABCD3* gene increased with increasing metastatic potential in a panel of prostate cancer cell lines derived from African American and Caucasian American men. Given importance of identifying biomarker(s) that can distinguish indolent versus aggressive prostate tumors, we conducted an immunohistochemical analysis of* ABCD3* expression Caucasian and African American prostate tumors.* ABCD3* expression in each patient population was compared with clinicopathologic characteristics, Gleason score, and age.* ABCD3* expression increased with increasing Gleason score (*P* = 0.0094), age (*P* = 0.0014), and pathology grade (*P* = 0.0007) in Caucasian patients. Interestingly, in the AA patients,* ABCD3* expression highly increased to the same degree in both low and high Gleason score tumors. Similarly,* ABCD3* expression was elevated to the same degree in BPH derived from AA. Our findings demonstrate that increased* ABCD3* expression correlates with Gleason Score in CA prostate tumors. However, in AA prostate tumors,* ABCD3* expression was higher and was sustained in both low Gleason and high Gleason AA tumors. While the functional role of* ABCD3* in prostate cancer is not completely elucidated, this gene warrants further study as a potential biomarker for aggressive prostate.

## 1. Introduction

Prostate cancer (CaP) is one of the most commonly diagnosed forms of cancer for men in the United States. An estimated 854,790 new CaP cases were projected for 2013 [1].

Even more alarming is the observation that African American men have both a higher risk and a higher rate of prostate cancer morbidity and mortality compared to men of other racial or ethnic groups in the USA and globally [1–3]. While there is evidence that the organ confined disease has similar outcomes for African American (AA) and Caucasian American (CA) men [4], AA prostate cancer patients develop clinical disease earlier [5] and appear to have the worst outcomes when the disease is diagnosed at advanced stages [4].

Though the major cause of CaP health disparity seen in AA men remains unclear, multiple studies show that genetic differences in AA and CA tumors play a major role. Wallace et al. [6] performed the first cDNA microarray study that identified differentially expressed genes in AA and CA with localized prostate disease. Their findings showed that several known metastasis associated genes, including* AMFR* (autocrine mobility factor receptor), chemokine (C-X-C motif) receptor 4, and matrix metalloproteinase 9, were more highly expressed in African Americans tumors. In addition, they found that a two-gene tumor signature,* PSPHL* and* CRYBB2*, accurately distinguishes prostate tumors in African Americans from tumors in European Americans. Importantly, Wallace et al. observed prominent differences in tumor immunology between AA and CA. We followed up this report using a similar cDNA microarray format, where we found 97 genes differentially expressed in AA tumors compared to CA tumors [7]. Interestingly, ontology enrichment of these differentially expressed genes included interleukins, progesterone receptor signaling, and chromatin modeling. Timofeeva et al. [8] conducted a cDNA microarray study in isolated epithelial cells derived from AA and CA patients. They found 67 genes differentially expressed in AA and 25 genes differentially expressed in CA. Seventeen differentially expressed AA genes were associated with metastasis, invasion, and migration. Of these 17 genes,* AMFR1*,* SOS1*, and* MTA2* were overexpressed in clinical prostate tumors. Careful validation of AA genes led to identification of* SOS1* as a potential candidate biomarker in AA men, consistent with the hypothesis that a biological basis exists for prostate cancer aggressiveness. Thus the preponderance of the data suggests that AA CaP patients have differentially expressed genes that could possibly contribute to the aggressiveness of CaP in AA.

To follow up on our cDNA microarray study [7], we performed genotype-phenotype, SNP, and expression transcript levels correlations using HapMap Yoruba population with our 97 differentially expressed genes in a SCAN database [9]. We found that two SNPs in* ABCD3* which strongly interact with the* RanGAP1* gene are important in AA prostate tumors. We confirmed this finding by monitoring* ABCD3* expression in a novel panel of African American [9] and Caucasian prostate cancer paired cell lines. The LNCaP, C4-2B, showed 2-fold increase; MDA-2PC-2B cell line, derived from AA, showed the highest fold-change, 10-fold. The EGFR overexpressing DU-145 WT cell line exhibited a 4-fold increase in expression relative to nontransfected DU-145 prostate cell lines. Furthermore, Ingenuity network analysis implicated that* ABCD3* is associated with either one, two, or three network hubs: ERK, MaPK, and NFkB pathways. It should be noted that other members of the ABC gene family, namely, ABCC3, ABCD1, and ABCD2, have been shown to confer chemoresistance in other cancer types, but, to our knowledge, we were the first to have reported an association of* ABCD3* with prostate cancer [9] as well as with prostate cancer health disparity [9].

*ABCD3*, a member of the ATP binding cassette or ABC family, encodes for PMP70, a 70 kdal peroxisomal membrane protein.* ABCD3* acts as an ATP-dependent pump that transports fatty acids into peroxisomes [10]. However there have been no reports to our knowledge of the* ABCD3* association with prostate cancer. Herein, we report that increased* ABCD3* expression correlates with prostate tumor aggressiveness; specifically increased* ABCD3* expression correlates with increasing Gleason score in CA patients. These CA patient findings prompted us to independently measure* ABCD3* expression in a small number (13) of AA prostate cancer patients. AA prostate tumors exhibit a high and sustained expression in both low Gleason and high Gleason tumors.* ABCD3* expression was also highly expressed in BPH from AA. Limitation of AA pilot study is the small sample size (13) of AA prostate cancer tissues used in this study and the absence of normal AA prostate tissue (negative CaP biopsy tissue). Future studies will include comparisons of AA and Caucasian prostate tumors (tumors and matched nontumor tissues).

## 2. Materials and Methods

### 2.1. Antibodies

Anti-*ABCD3*, a polyclonal antibody produced in rabbit, was purchased from Aldrich Sigma Chemical Company (St. Louis, MO; catalog number HPA032027).

### 2.2. Prostate Tissue Specimen

The use of all tissues was approved by the Institutional Review Boards of Florida A&M University and Tuskegee University and University of Hawaii Committee on Human Studies. Prostate tissue microarrays (TMA) were obtained from a commercial supplier (US Biomax, Rockville, MD; TMA catalog number PR2085b). The TMA (PR2085b) contained biospecimen from 114 patients consisting of 92 adenocarcinomas, 2 prostate transitional cell carcinomas, 12 prostate adjacent normal tissues, and 8 normal prostate tissues, with duplicate cores per prostate cancer patient and single core per patient of adjacent normal and normal tissue. The array format was in duplicate cores per patient. The tissue samples were formalin fixed, paraffin embedded. Tissue array sections were mounted on the positive charged SuperFrost Plus glass slide. The tissue microarray sections were cut at 5 microns in thickness. Individual cores were 1.0 mm in diameter and were spaced 0.25 mm, and US Biomax supplied the following clinicopathologic characteristics of the subjects whose tissue was on the TMA: gender, age, grade, Gleason grade, Gleason score, and TNM staging. Tissue from this array represents the Caucasian population as US Biomax could not confirm that AA tissues were included on the TMA.

Population-based archival prostate cancer specimens were identified from Surveillance, Epidemiology, and End Results (SEER) Tissue Repositories in Hawaii [11, 12]. As this was a retrospective study using archive tissue specimens and State of Hawaii cancer registry data, the IRB waived the need for written informed consent. Formalin-fixed paraffin-embedded (FFPE) tumor specimens from African American patients with primary and aggressive prostate adenocarcinomas as well as BPH were obtained from the SEER Residual Tissue Repository (RTR) of the University of Hawaii Cancer Center. These samples were derived from cancer cases from the years 1986 to 2009. These de-identified samples contained clinical and pathological annotated data collected by the SEER program member registries in Hawaii.

### 2.3. Immunohistochemical Staining

Immunohistochemical analysis of* ABCD3* expression was performed in two independent laboratories (AA samples were accessed from the SEER RTR in Hawaii and analyzed in the University of Hawaii Cancer Center Pathology Resource Laboratory and the prostate tissue microarray samples were accessed and analyzed in the US Biomax Laboratory, Rockville, MD, USA). All prostate tissues were subjected to immunohistochemical (IHC) analysis using a polyclonal antibody for* ABCD3* (Sigma, St. Louis, MO). Prostate tissues were deparaffinized in xylene and rehydrated in graded alcohols. Antigen retrieval was performed before incubating with primary antibody. Antigen retrieval of* ABCD3* was accomplished by using 1x antigen retrieval solution, 20 minutes in microwave oven with simmering conditions. Slides were cooled at room temperature for 15 minutes. Endogenous peroxide activity was quenched with 3% hydrogen peroxide for 5 minutes. Slides were blocked with 2.5% normal horse blocking serum and incubated at room temperature for one hour in humidity chambers with* ABCD3* primary antibody diluents. After several washes, slides were incubated with ImmPRESS reagent anti-rabbit Ig (peroxidase), catalog number MP7401/Vector Laboratories, for 30 minutes. The antigen-antibody reaction was visualized after a 10-minute incubation in DAB solution. Positive controls were included in each staining run; negative controls were obtained by omitting the primary antibody. Slides were dehydrated in alcohols and cleared in three xylene baths before being mounted with permanent mounting media. Slides were counterstained with Hematoxylin QS (Vector Labs, H-3404).

TMA and formalin-fixed paraffin-embedded (FFPE) tumor specimens were scored for membranous, cytoplasmic, and/or nuclear staining for* ABCD3* and classified with respect to the intensity of immunostaining. The* ABCD3* expression levels in prostate tissue on the TMA were classified as negative (≤0.3), weak positive (0.3 to 1.0), or strong positive (≥1.5).

### 2.4. Statistical Analyses

All data presented as means ± SD were analyzed with Prism software (GraphPad Prism version 5; La Jolla, CA). The significance of the observed differences was determined with Student's *t*-test, Pearson *χ* ^2^ test, or Fisher's exact test.

## 3. Results

### 3.1. Increased* ABCD3* Expression Correlates with Gleason Score in Caucasian American Patients

**Figure 1:**
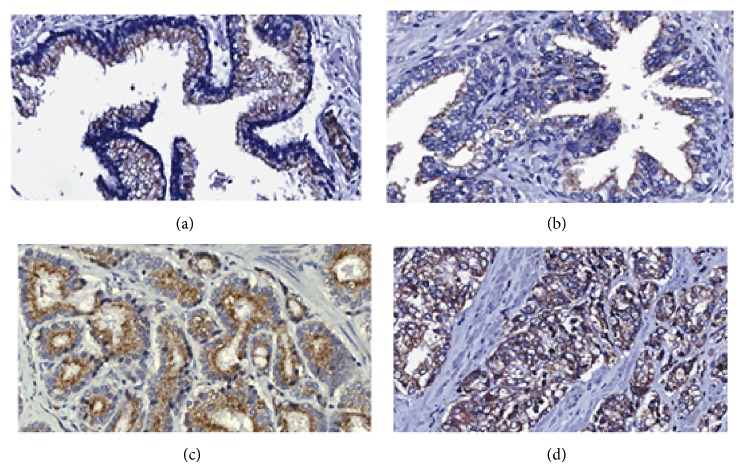
Detection of* ABCD3* immunostaining in Caucasian prostate tumors. The anti-*ABCD3* antibody staining was predominantly in the peroxisomal membrane in normal and adjacent normal tissues (Figure 1).* ABCD3* is mildly expressed in normal/normal adjacent human prostate tissue (Figures 1(a) and 1(b), resp.).* ABCD3* staining is moderate to strongly intense stained in malignant (low Gleason and high Gleason) prostate tissues (Figures 1(c) and 1(d), resp.). 20x magnification of representative images from prostate tissue microarray is shown.

To evaluate the expression of* ABCD3* during prostate cancer progression, immunohistochemistry was conducted in Caucasian American* ABCD3* expression in 206 cores from 114 Caucasian patients, including normal tissue (20 patients), low Gleason tumors (87 patients), and high Gleason tumors (81 patients).* ABCD3* expression was detected using immunohistochemical staining with an anti-*ABCD3*, a polyclonal antibody; the intensity of brown staining corresponds to the level of* ABCD3* expression.  Figure 1 shows representative images of* ABCD3* staining in* normal (Figure 1(a)), normal adjacent (Figure 1(b)), low Gleason (Figure 1(c)), and high Gleason (Figure 1(d)) prostate tissues* in Caucasian American males.

*ABCD3* staining was observed predominantly in the peroxisomal membrane of noncancerous and prostate cancer tissues, which was expected because the* ABCD3* gene product, PMP70, is a membrane bound peroxisomal protein. Malignant prostate tissue showed increased* ABCD3* expression in the peroxisomal membrane, with punctate staining in the cytoplasm. The relative distribution of staining intensity is summarized numerically in Table 1.

Numerical analysis of* ABCD3* across Caucasian prostate cancer progression is given in Table 1.* ABCD3* stain intensities ranged from 0 to 1.5 in Caucasian prostate tumors.* ABCD3* expression was weak in normal human prostate tissues and moderate to strong in malignant prostate tissue with low and high Gleason scores (43 out of 87 low Gleason tissues stained moderate-to-strong while 36 out of 87 high Gleason tissues showed strong staining) (see Table 1).

**Figure 2:**
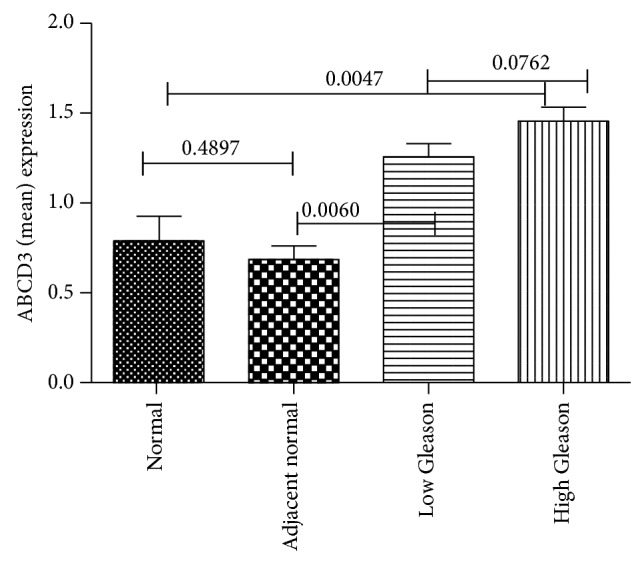
Graphical depiction of* ABCD3* expression in normal, low, and high Gleason Caucasian prostate tissues.* ABCD3* expression was weak in normal human prostate tissues and moderate to strong in malignant prostate tissue with low Gleason and high Gleason score tumors.

The overall distribution of* ABCD3* staining intensity* indicated strong ABCD3 staining in both low Gleason score and high Gleason score prostate tumors relative to noncancerous tissue as indicated in Table 1. *In Figure 2, a graphical representation of* ABCD3* staining in Caucasian patients showed no statistically significant difference between normal and normal adjacent tissues (*P* = 0.4897).

However, the difference in* ABCD3* expression between normal and high Gleason was statistically significant (*P* = 0.0047). Similarly, the difference in increased* ABCD3* expression between low Gleason and adjacent normal tissues was statistically significant (*P* = 0.0060). Correlation of* ABCD3* expression with clinicopathological features showed a positive correlation with patients' age (*P* = 0.0018), with Gleason score (*P* = 0.0127), and with well-to-moderately differentiated and poorly differentiated prostate tumors (*P* = 0.0009). A comparison of low grade tumors revealed that* ABCD3* expression did not have a significant correlation (data not shown). Taken together, these data indicate that increased* ABCD3* expression correlates with severity of prostate cancer differentiation in CA patients.

### 3.2. *ABCD3* Expression in African American Prostate Patients Exhibits a Sustained Elevation with Prostate Cancer Progression

**Figure 3:**
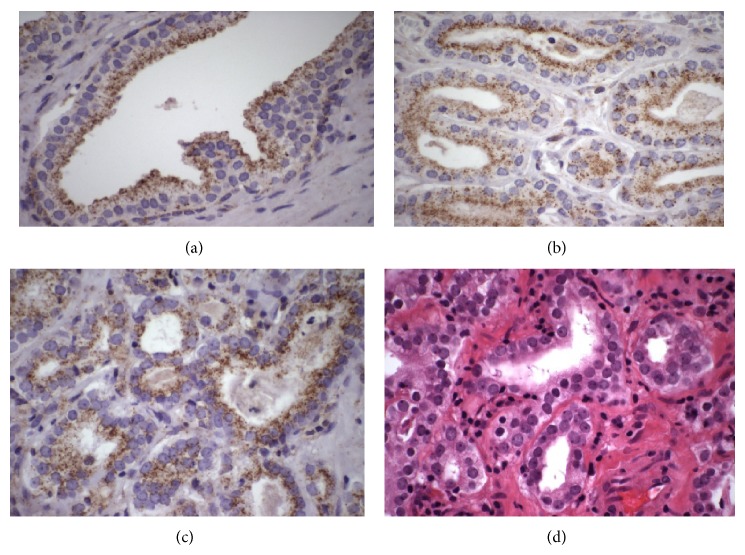
Detection of* ABCD3* immunostaining in African American BPH and prostate tumors. A representative image of BPH from AA is shown in (a), while (b) and (c) are representative images of low Gleason tumors and high Gleason tumors, respectively.  Figure 3(d) shows H&E staining in high Gleason tumor.* ABCD3* staining was very strong in BPH and low and high Gleason tumors. Staining appeared in the peroxisomal membrane. We also observed staining in what appeared to be blebbing of peroxisomal membranes.* ABCD3* staining pooled in the cytoplasm. Low Gleason tumors exhibited strong (≥3) staining in the peroxisomal membrane and strong (≥3) staining in glandular epithelial cells and in the stroma. Representative image of high Gleason tumor shown in (c) also exhibited strong* ABCD3* staining (≥3) in blebbing peroxisomal membranes, with sporadic* ABCD3* pooling in the cytoplasm and staining in the stroma. All images shown are at 600x magnification. A representative image of low Gleason tumor (Gleason 5-6; see Figure 3(b)) exhibited strong staining in the peroxisomal membrane and strong staining in glandular epithelial cells and in the stroma. High Gleason score tumors (Gleason 7-8; see Figure 3(c)) also exhibited strong* ABCD3* staining (>3) with noticeable blebbing in the peroxisomal membranes, random* ABCD3* pooling in the cytoplasm, and staining in the stroma. Scoring for* ABCD3* staining intensities ranged from 0 to 3.

TMA findings for CA prostate cancer patients in this study prompted us to evaluate* ABCD3* expression in the limited number of AA prostate tumors available to us. Previously, we reported that* ABCD3* expression was associated with AA CaP patients [7, 9]. Therefore, we next sought to determine if there is differential expression of* ABCD3* in AA prostate tumors. Thirteen AA CaP patients with representative biospecimen from BPH, low Gleason tumors, and high Gleason tumors were analyzed for* ABCD3* expression by IHC as described above. In BPH (Figure 3(a))* ABCD3* was strongly expressed in what appeared to be blebbing peroxisomal membranes and some* ABCD3* staining was pooled in the cytoplasm.

*Correlation of Overall ABCD3 Expression with Clinicopathological Features of CA Prostate Cancer Patients*. The correlation between* ABCD3* expression and clinicopathological features is summarized in Table 1. Statistically, the mean expression of* ABCD3* was significantly associated with age at ≤ 67 (*P* = 0.0014), with well-to-moderately and poorly differentiated prostate tumors (*P* = 0.0009), and with Gleason score of ≤6 (*P* = 0.0094). This is the first report of* ABCD3* expression being statistically significantly associated with Gleason score, age, and severity of differentiation in CA prostate tumors (see Table 2).

## 4. Discussion

We are the first to report that* ABCD3* is associated with prostate cancer [9]. This study is a follow-up investigation to determine if* ABCD3* can be detected in human prostate tumors. Herein we observed immunohistochemical staining of* ABCD3* to be localized predominately in the peroxisomal membrane, with some evidence of* ABCD3* cytoplasmic pooling during prostate cancer progression. Our studies revealed that increased* ABCD3* expression correlates with Gleason score in the Caucasian patient population. In a separate study, we measured* ABCD3* expression in African American prostate patients and observed a sustained elevation of* ABCD3* in low Gleason score through high Gleason score in AA prostate tumors. Furthermore, elevated* ABCD3* expression in the noncancerous BPH tissues from African American men was surprising, since BPH is not cancerous tissue.

The observation that* ABCD3* was highly overexpressed in African American patients was intriguing, even though the biological consequences of* ABCD3* overexpression in prostate tissue are not yet clearly understood. We know of several independent reports that have identified overexpressed proteins in African American prostate cancer patients. One such overexpressed protein, in African American prostate cancer patients, is the epidermal growth factor receptor, EGFR [13]. Also noteworthy is* SOS1*, a regulator of EGFR expression and downstream signaling, which also shows increased expression in African American prostate cancer patients [8]. More recently, Kaiso was shown to be overexpressed in AA prostate tissues as well [14]. Importantly, Kaiso expression was positively influenced by EGFR activation, which led to the speculation that overexpression of EGFR contributes to increased Kaiso levels in the AA patient population. It is fascinating that* SOS1* and Kaiso, two genes overexpressed in AA, are also associated with EGFR and this knowledge provides a sound basis for initiating studies that look at the interdependence of* ABCD3* expression and EGFR activation/expression. In addition, the interdependence of* ABCD3*,* SOS1*, and Kaiso expression in AA prostate tissues will be explored for insight into how overexpression of* ABCD3* plays a role in the aggressive prostate cancer disease seen in African American prostate cancer patients relative to Caucasian patients in epidemiologic studies [15, 16]. In silico data from our previous report [9] implicated that the* ABCD3* gene may be regulated by one of three or all three network hubs, ERK, Mapk, and NFkB pathways. Of these three hubs, the MapK/ERK pathway most probably plays a significant role in regulating* ABCD3* expression for two reasons. The first is that activation of the Mapk/ERK pathway via EGFR stimulation is vital for increased transcription of numerous cancer related genes [13]. Hence, it is rational to speculate that MAPK/ERK pathway via EGFR stimulation may contribute to modulation of* ABCD3* gene expression in prostate cancer. Secondly, the EGFR overexpressing DU-145 WT cell line exhibited a 4-fold increase in* ABCD3* expression relative to nontransfected DU-145 prostate cell lines [9]. Collectively these findings seem to be sound premises to initiate studies that will test the hypothesis that activation of EGFR stimulation contributes to increased* ABCD3* expression.

## 5. Conclusions

In summary,* ABCD3* expression correlates with severity of prostate cancer differentiation in the Caucasian prostate cancer patient. In a separate, small scale study of low and high Gleason African American prostate tumors,* ABCD3* expression was elevated and sustained* ABCD3* overexpression in primary, low, and high Gleason tissues strongly suggests that prostate cancer is a more aggressive disease in African Americans. Limitations of the studies done with African American tumors are the small sample size and the inability to obtain nontumor control prostate tissues for AA. Future studies will include a comparison of AA and Caucasian prostate tumors and their matched nontumor tissues of the same stage and grade for each racial group.

Moreover, additional studies are needed to define the consequences of* ABCD3* overexpression in AA prostate tumors; however due to the intrinsic role of* ABCD3* in fatty acid beta oxidation, this would imply that* ABCD3* could have a role in enhanced growth in both BPH and malignant cells. Hence its oncogenic role in tumor development and progression, must be extensively investigated and* ABCD3* should be investigated and validated as potential biomarker that can distinguish indolent tumors from those that will go on to become metastatic.

## Figures and Tables

**Table 1:** Overall ABCD3 expression in normal, low, and high Gleason score prostate carcinomas.

* ABCD3*	Normal^*^ *N* = 20	Low Gleason *N* = 87	High Gleason *N* = 81
No score (0)		1	4
Weak (≥1)	18	43	41
Moderate (≥2)	2	31	23
Strong (≥3)		12	13

^*^includes adjacent normal.

**Table 2:** Correlation of *ABCD3* overall expression with clinical features.

Characteristics	All patients	Overall expression		*P* ^†^
		≥1.5 (median)	<1.51	
Total	**142**	**70**	**72**	
Age				
≥66 (median)	71	49	38	0.0018
<66	71	94	27	
Grade (differentiation)				
Well-moderate	70	54	35	
Poorly undifferentiated	69	28	54	0.0009
Gleason score				
≥5	52	27	27	
	50	77	31	0.0127

^†^ *P* value for the correlation of mean expression with clinical feature. *P* values were obtained with the *χ* ^2^ test.
